# Supplementary material for: Clinical and Genomic Evaluation of 207 Genetic Myopathies in the Indian Subcontinent
Source: Front Neurol. 2020 Nov 5;11:559327. doi: 10.3389/fneur.2020.559327 (PMC7674836; doi:10.3389/fneur.2020.559327)
Supplement: Supplementary file 1 [file Table_1.DOCX]

Supplementary Material

# Supplementary Methods:

**Questionnaires for patient recruitment:** Patient recruitment questionnaires for recruitment to the sequencing program are given below.

1. Is there anyone in the patient’s families with similar symptoms? If so, are their symptoms the same, milder, or more severe than the patient’s?

NOTE: list all family members and how their symptoms compare

- Brother

- Sister

- Father

- Mother

- Son

- Daughter

- Maternal Uncle

- Other aunt or Uncle

- Male first cousin

- Female first cousin

- Other

2. Ancestry (select all that apply)

- Finnish

- Other Northern European

- Japanese

- Not listed

- Unknown

3. Have any LGMD subtypes or other conditions been ruled out for this patient by genetic testing? NOTE: It is recommended that you initially run the tool without any exclusions

- Yes

- No

- Unknown

If yes, check all the diseases that apply

2A 2B SG's 2G 2H 2I 2J DG's 2L 2Q

1A 1B 1C 1D 1E 1F 1G 1H Nonaka/HIBM

Tibal Becker DMD Manifesting Carrier FSH EDMD

Pompe Bethlem

4. Age of onset of symptoms

NOTE: Onset of symptoms should not include high CK without weakness symptoms or subclinical changes e.g. on MRI.

- <5 years

- 5-12

- 13-25

- >25

- Unknown

5. If you had an electromyogram (EMG), what were the results? This is the test in which needles are stuck into muscles to record nerve impulses. Go to http://www.nlm.nih.gov/medlineplus/ency/article/003929.htm for a description of EMG

 The EMG showed a muscle problem (myopathic pattern)

 The EMG showed a nerve problem (neurogenic pattern)

 I have never had an EMG or I don't remember the results

6. Distal Weakness or Proximal Weakness?

- Primarily Distal

- Comparable to or less than proximal weakness

- None, or much less than proximal weakness with much later onset

- Unknown

7. Facial weakness

NOTE: Examples of this could be difficulty chewing, whistling, making normal facial expression. Also, onset is particularly early in the disease course prior to having severe generalized weakness.

- Yes

- No

- Unknown

8. Did you have nervous system symptoms (for example: headaches, tiredness, sleep disorders) which began within one year of the onset of muscle weakness? If yes, do you have respiratory difficulties?

 I have some of these symptoms, and I have respiratory difficulties.

 I have some of these symptoms, but I do not have respiratory difficulties.

 I do not have any of these symptoms.

 I don't know

9. Scapular Winging

NOTE: If the answer is NO, then you need to determine for how long they have had muscular dystrophy symptoms. If it has been for less than a couple of years, then the answer should be UNKNOWN because we don’t know how soon patients start to show scapular winging

- Pronounced

- Mild

- No

- Unknown

10. Calf Hypertrophy

Note: Answer PRONOUNCED or MILD if calf muscles were enlarged earlier in the disease course, even if they aren’t now

- Pronounced

- Mild

- No

- Unknown

13. Pain/Cramps

11. Asymmetry of Weakness/Wasting

- Pronounced (persistent)

- Mild

- No

- Unknown

12. Rate of progression

NOTE: descriptions of each rate can be found below

- Rapid: significant increase in weakness within a few years, causing loss of function and/or walking and/or involvement of other muscle groups beyond the first ones affected.

- Moderate: increase in weakness over several years, but only a slight increase in weakness from year to year. No loss of walking or great difficulty walking within 10 years of onset.

- Slow: very gradual or no decrease in strength for several years following diagnosis. Function appears stable or nearly so.

- Unknown: answer this if a detailed history isn’t available, or if patient has only recently experienced symptoms (<3 years) and there has not been noticeable progression.

13. Biopsy: Dystrophic

NOTE: Does the patient’s muscle biopsy show a dystrophic appearance (such as fiber size variation, central nucleation, focal necrosis, fiber replacement by fatty or connective tissue)?

- Yes

- No

- Unknown

14. Biopsy: Inflammation –

Note: Does patient’s biopsy show inflammatory characteristics (e.g., substantial levels of mononuclear cell infiltration?

- Yes

- No

- Unknown

15. Biopsy: Vacuoles/Inclusion Bodies –

NOTE: Are rimmed vacuoles observed (in substantial numbers) on patient’s biopsy?

- Yes

- No

- Unknown

16. CK results

NOTE: If the patient has more than one CK measurement, use the one taken closest to the onset of the patient’s muscle symptoms

- Unknown/Not Measured

- <200

- 200-500

- 500-2000

- 2000-7000

- >7000

17. Does the patient’s biopsy show aggregation of desmin or other myofibrillar proteins (conditions with this biopsy appearance are often referred to as a Myofibrillar Myopathy)?

- Yes

- No

- Unknown

18. Finger contractures

- Yes

- No

- Unknown

19. Toe walking/Achilles tendon tightness

- Yes

- No

- Unknown

20. Foot drop

Do you trip often or has the doctor ever said you have a foot drop?

Explanation: This is where you trip over your own foot because you can’t pull your foot through the walking motion (lack of dorsiflexion).

- Yes

- No

- Unknown

21. Inability to stand on toes

- Unable

- Able

- Unknown

22. Quadricep Strength (early in disease course if patient now has severe generalized weakness)

- More affected than other proximal leg muscles

- Affected comparably to other proximal leg muscles

- Selectively spared compared to other proximal leg muscles

- Unknown

23. Neck weakness

- Yes

- No

- Unknown

24. Legs or arms more affected

- Legs

- Arms

- Arms and legs comparable

- Unknown

25. Cardiac conduction defect

Do you have a pacemaker, or abnormal heart rhythm?

- Yes

- No

- Unknown

26. Dilated or Hypertrophic Cardiomyopathy

Has your doctor ever told you that you have a “dilated cardiomyopathy” or “hypertrophic cardiomyopathy”?

- Yes (dilated)

- Yes (hypertrophic)

- No

- Unknown
